# Beef Quality Preferences: Factors Driving Consumer Satisfaction

**DOI:** 10.3390/foods9030289

**Published:** 2020-03-04

**Authors:** Chad Felderhoff, Conrad Lyford, Jaime Malaga, Rod Polkinghorne, Chance Brooks, Andrea Garmyn, Mark Miller

**Affiliations:** 1 Department of Agricultural and Applied Economics, Texas Tech University, Lubbock, TX 79409, USA; chad@muenstermilling.com (C.F.); Conrad.Lyford@ttu.edu (C.L.); Jaime.Malaga@ttu.edu (J.M.); 2 Birkenwood Pty. Ltd., 46 Church St, Hawthorn, VIC 3122, Australia; rod.polkinghorne@gmail.com; 3 Department of Animal and Food Sciences, Texas Tech University, Lubbock, TX 79409, USA; chance.brooks@ttu.edu (C.B.); andrea.garmyn@ttu.edu (A.G.)

**Keywords:** beef, consumer, demographics, eating quality, flavor, satisfaction, tenderness

## Abstract

The current study was designed to broaden the understanding of the attributes impacting the sensory properties of beef when consumed. Using a survey of consumers from three different geographical regions in the United States (US), we determined the impacts of three attributes on overall satisfaction in several different ways. The two main statistical methods used were an Ordinary Least Squares (OLS) model and the Conditional Logit model. Perhaps the most important finding of this study was that flavor was the largest contributor to consumer satisfaction. This finding was consistent throughout all the models. In the base model, flavor represented 59% of the satisfaction rating. Additionally, results indicated domestic beef was preferred over Australian beef by US consumers. Another important finding of the study was the impact of the demographic variables of age, income, and gender on satisfaction. The older group generally placed more emphasis on tenderness, while younger people preferred juicier beef. Males were more responsive than females for all attributes, especially tenderness. Those with higher income were more responsive to tenderness for all quality levels, but the lower income group was more responsive to juiciness. Overall, flavor had the largest impact on consumers’ satisfaction level in comparison to tenderness or juiciness.

## 1. Introduction

The beef market has always been under constant pressure of evolving preferences in areas such as taste, consistency, and healthfulness [1,2,3]. Differences in consumer lifestyles and their impacts on product selection are well recognized by processors and marketers of food [4,5]. Transformation in the demand structure presents many significant opportunities for beef producers and marketers. This changing demand structure signifies an important move from beef being marketed primarily as a homogenous commodity to a niche product [6]. 

Historically, the lack of ability by marketers to respond to the changing environment in the marketplace is exposed by the decrease in consumption. The United States Department of Agriculture (USDA) [7] has estimated that beef consumption along with other red meats has declined for the last several decades, based on per capita availability. This creates substantial concern within the beef production–marketing system. Changes in the pricing structure of competing meats alone cannot explain the shifts in beef demand [8]. 

Previous studies have identified beef characteristics that are perceived as desirable with the goal of increasing demand. In many cases researchers have focused on the tenderness attribute of beef in a retail environment [6,9,10,11]. The retail market is commonly believed to be the end market for beef; hence, researchers typically focus their efforts on understanding the consumer purchase decision at the retail level. External factors present in the retail environment influence the consumers’ perception of quality, suggesting that this environment may add an element of bias to studies [12]. The current study controls for these external factors by using an untrained sensory test outside of the retail or home environment to evaluate fundamental quality preferences in beef.

We look to answer many questions about consumers’ preferences by using consumer choice modeling. The consumer choice model allows consumers to make decisions about products based on several key attributes [6]. The developed model evaluates the consumer rating of chosen attributes and their effects on consumer satisfaction by using a large scale study. 

The overall focus of this study was to evaluate the extent to which United States (US) beef consumers vary in their preference. Multiple objectives arose, including determination of the impact of tenderness, juiciness, and flavor on consumers’ overall satisfaction and what changes were required to increase consumer satisfaction to higher perceived quality levels. Moreover, we wanted to estimate the quality attributes that correspond with distinct levels of satisfaction, as well as evaluate the effect of the beef products’ country of origin on consumer satisfaction. Our final objective was to determine if consumers’ preference structures vary by demographic characteristics. 

## 2. Materials and Methods

### 2.1. Sampling Methodology

Methods of gathering data in previous studies have varied widely, were generally limited to a smaller scope (less than 1000 consumers), and were less representative of the overall US population. Data in the current study were gathered by using the combination of a survey and untrained sensory tests in three US cities (*n* = 1440; 480/city). These data originated from the following diverse metropolitan areas across the US: (a) Phoenix, AZ, (b) Lubbock, TX and (c) Washington, DC/Baltimore, MD. These cities were chosen based on the results from a previous study conducted as representative of the overall US population [11]. Phoenix was chosen because of its diverse population and proximity to the western side of the US. Lubbock was used to represent the central region of the US and because Miller et al. [11] showed the preferences of beef consumers in Lubbock, TX, were not different from those of beef consumers in Dallas, TX. The Washington, DC/Baltimore area was chosen because of its diverse population and proximity to the east coast. 

There were essentially two parts to the data collected in this study: survey data and untrained sensory results. However, this study focused on the demographic characteristics of the consumer along with the untrained sensory test results. The demographics were gathered from consumer responses to predetermined demographical questions on the survey. 

The use of the untrained sensory test allowed for the gathering of measurements that lead to the discovery of the consumer’s fundamental beef preference. In previous consumer studies, the cut (muscle) and origin (country and/or feeding system) were made known to the panelist [9,13]. This can present a problem because consumers have been shown to rank the cut of meat as the most important factor in determining beef quality [12]. The cut, along with other outside factors, can create biases in the consumers’ perception of quality. Moreover, Ron et al. [14] found the consumers’ perception of eating quality can be influenced by revealing quality-differentiated brand names and labeling claims to the consumer, particularly claims related to production practices. It was the above reasons that influenced us to use untrained “blind” sensory tests which control for specific external factors.

### 2.2. Product

One major variable in our study was the origin of the sample. The origin refers to the muscle being tested as well as the country and/or feeding system that the beef was generated from. Four subprimals were used in this study: outside round, *semimembranosus*; top sirloin butt, *gluteus medius*; tenderloin, *psoas major*; and strip loin, *longissimus lumborum*. Australian product was collected from cattle that were grass-fed, short fed grain for 70 days, or long fed with grain for 188 days. All grain-fed cattle had received a hormonal growth promotant (HGP) implant, whereas none of the grass-fed cattle were implanted. HGP usage was monitored and reported for Australian cattle when they were transferred to a Meat Standards Australia (MSA) licensed abattoir. Live animal information pertaining to diet (grass vs. grain) was made available through the animal identification system in Australia, but no data were collected on-farm. 

Sixty carcasses (*n* = 20/feeding type) were selected in Australia at two Queensland abattoirs. Forty carcasses (grass and short fed grain) were obtained from one abattoir and 20 carcasses (long fed grain) were obtained from the second abattoir. US product was sourced from Select and High Choice USDA graded carcasses at a commercial abattoir in Nebraska. The US carcasses (*n* = 60; 30/quality grade) were selected to evenly represent each USDA grade, whereas the Australian carcasses were taken as a consecutive run from each category. US cattle were all commerically grain-fed. According to cattle feeding surveys, feedyard finishing rations in Nebraska consist predominately of corn, distillers grains, haw and straw, silage, and mineral [15]. Cattle in the Northern Plains (Nebraska, South Dakota, North Dakota) are on finishing rations an average of 137 d [15]. It was likely cattle had received at least one implant as 95% of cattle in this region reportedly receive some type of growth enhancing technology and over 80% of feedlots in the Northern Plains (including Nebraska) report using implants. However, exact duration of the finishing ration and actual HGP usage was not known in the current study due to their selection from a commercial abattoir. All carcasses were graded by a common MSA grader with the US carcasses also assessed by a senior USDA grader. 

### 2.3. Preparation

Subprimals were fabricated into 2.5 cm steaks, and further processed into 2.5 × 5 × 5 cm steak pieces and vacuum packaged at 10 mbars of pressure (Cryovac barrier bag; moisture vapor transmission rate: 0.3 to 0.6 g/100 in^2^/24 h; oxygen transmission rate: 1.5 to 3.5 cc/m^2^/24 h; Sealed Air Food Care; Charlotte, NC, USA) as sets of five steak pieces in sequential order according to anatomical position. All samples were frozen (−10 °C) at 14 days postmortem, so that aging period did not differ between samples. The frozen steak pieces were sorted into a predetermined cook order. Steak preparation from the primal cuts, allocation to cooking order, and consumer allocation followed the MSA protocols [16].

Samples were thawed at 2–4 °C for 24 h prior to consumer panel evaluation. All samples were prepared on a Model S-143 K Silex clamshell grill (Silex Grills Australia Pty Ltd., Marrickville, Australia) with plate temperature set at 225 °C. A strict time schedule was used to ensure all steaks were prepared identically [16]. Ten sample steaks were prepared on the grill for each cooking round. All steaks were cooked for 5 min and 45 s. After a mandatory 3 min rest period, each steak was cut in half into two equally sized rectangular pieces and served to two separate preselected consumers. 

### 2.4. Consumer Panels

The Texas Tech University Institutional Review Board approved procedures for use of human subjects for consumer panel evaluation of sensory attributes. The survey team provided a monetary incentive to local organizations for providing volunteers, which formed the consumer groups. The groups then met at a specified location to participate in the sensory test. Consumer panelists were only allowed to participate once. 

The sensory test was administered on eight different nights to groups of sixty volunteers in each of the three cities. Consumers were asked to fill out a survey regarding demographics and prior beef preferences. The summary statistics for demographics of the population can be found in Table 1. 

Each panelist was seated at a numbered consumer booth and provided a ballot, plastic utensils, toothpicks, a napkin, an expectorant cup, a cup of water, and palate cleansers to use between samples (unsalted crackers and a 10% apple juice, 90% water solution). Prior to the start of each panel, panelists were given verbal instructions about the ballot and the procedure for the testing of samples. Panelists were instructed to cut each sample using their utensils to a size representative of beef consumed in the home or restaurant. The panels were conducted in large rooms with tables that had been divided into individual sensory booths.

Eight notional products of differing quality were designated by the combination of four cuts and two USDA grades. Australian samples were allocated to the assumed closest USDA grade. Each consumer was served six of the eight products with three samples being drawn from Australian sourced product and three from US sourced product. The six products used were balanced to ensure that each product was tested an equal number of times in each US city. In addition, each of the six individual products were allocated by a Latin square design which balanced presentational order. Consumers were served a total of seven samples in a predetermined balanced order in accordance with a 6 × 6 Latin square. All consumers received a warm-up sample to orient them to the sample format and evaluation procedures. Data obtained from warm-up samples was excluded from the analysis, as these samples were not related to the trial. The warm-up samples were always served in the first position, followed by six test samples. This design provided a balance for frequency, order, and carryover effects [17]. All samples were identified with a unique identification code assigned by the MSA software [16]. Each sample was rated on a 100 mm continuous line scale for tenderness, juiciness, flavor, and overall liking. On the scale, 0 mm was verbally anchored as not tender, not juicy, dislike flavor extremely, and dislike overall extremely, and 100 mm was verbally anchored as very tender, very juicy, like flavor extremely, and like overall extremely. Additionally, consumers rated each sample as “unsatisfactory”, “good everyday quality”, “better than everyday quality”, or “premium quality.”

In this study, consumer satisfaction was measured for the cut of meat and was the dependent variable for all models in this study. There were two types of satisfaction. One was a discrete choice while the other was measured on a continuum. The continuum measurement was the consumer’s perceived satisfaction on a 0 (worst) to 100 (best) scale. Discrete satisfaction levels were identified by the level of quality at which a consumer makes an acceptability decision, as follows: (2) “unsatisfactory”; (3) “good everyday quality”; (4) “better than everyday quality”; and (5) “premium quality.” For the purposes of this study, many parameters were presented as having an impact on overall satisfaction. The attributes of focus for this study were tenderness, flavor, and juiciness, all of which were measured from 0 to 100. These attributes were initially assumed to have a diminishing marginal utility as it can generally be expected that as satisfaction rises additional increases in attributes have declining effects. Overall, we expected these attributes had a positive influence on satisfaction. In addition, four different sources of beef were used in this study: Australian grass-fed beef, Australian grain-fed beef, USDA Select, and USDA Choice. These were represented as dummy variables in the model. 

### 2.5. Conceptual Framework

Given the panel nature of the data, there were two problem areas that could arise and decrease the accuracy of the results. One area was heteroskedascity, which was tested for by using White’s test and corrected where found. 

The second problem area was nonrepresentative samples. As is common in empirical marketing, it is important to eliminate nonrepresentative observations. Such observations typically reduce the effectiveness in estimating the model. The method chosen to correct for these observations was the Cook’s Distance method. Cook’s Distance (Di) is identified by the Equation (1) [18]:

(1) $$D_{i}=\frac{\sum_{j=1}^{n}{\left(y_{j}-y_{j}(i)\right)}^{2}}{(k+1)s_{2}},i=1,\ldots ,n$$

where: *s* = estimated root mean square error, *y_j_ =* regression estimate of the conditional mean *E*(*Y_j_*|*x*_1*j*_,…,*x_kj_*), and *y_j_(i*) = regression estimate of the condition mean *E*(*Y_j_*|*x*_1*j*_,…,*x_kj_*) with the *i*th data point (*y_i_,x*_1*i*_,…,*x_kj_*) removed.

This produces a normalized measure of the influence of point *i* on all predicted mean values. A common rule of thumb is to treat any point *i* as an outlier when the value of (*D_i_*) exceeds $\frac{4}{n-(k+1)}$ where *n* = number of observations and *k* = degrees of freedom. The use of Cook’s Distance increases the efficiency of the model and increases *R*^2^. The Cook’s Distance procedure was used in the data for all models of this paper. The scatter plots that depict the relation of flavor to satisfaction show the effects of using Cook’s Distance (Figures S1 and S2 for raw and cleaned, respectively). As one can see, the scatter plot narrows when the outliers were removed from the equation. The same can be seen for tenderness (Figures S3 and S4 for raw and cleaned, respectively) and juiciness (Figures S5 and S6 for raw and cleaned, respectively).

Marketers are continually looking for ways to better meet consumer preferences. An important key in understanding the preferences of the consumer is their experience while eating the product. From the evaluation of the consumers’ reactions, marketers can then develop products with the appropriate attributes that can closely fit the preferences of consumers. In addition, the preference structure provides marketers with a method for making assumptions about populations that contain the same characteristics. 

The food choice process is influenced by a large number of complex factors, including the person making the decision and the associated environment [12]. Product characteristics such as quality, price, and usefulness are among the common factors believed to influence the consumers’ purchase decision. The relevance of product characteristics to the individual consumer lies in their ability to generate some response (positive or negative), relative to a consumer’s perception of quality and ultimately the purchase decision [5]. Characteristics generating positive responses to quality perception are considered to add to utility gained by the consumption of the product. 

The preference structure of the consumer is revealed by several methods. The basic concept for these methods is to measure the reaction of consumers to changing attribute levels. Commonly used methods include surveys, focus groups, feedback, and consumer trials. These methods are specifically designed to evaluate the interaction of the internal and external factors that influence the purchase decision. Internal factors refer to the product attributes while the external factors refer to consumer demographics and market characteristics such as labeling and stores. Attribute values are assigned by the consumers as a reflection of the satisfaction gained from the product. Factors such as age, income, and education level have been shown to influence the consumers’ purchase decision [6,19,20]. In the current study, these factors were included in our model to capture their impact on satisfaction.

To better understand the impact of the above factors on satisfaction, we must first understand their interaction with consumer satisfaction. The interaction can be best explained by choice theory. Modern economic choice theory starts with the assumption that an individual’s market behavior is generated by maximization of utility [21]. According to consumer choice theory, a person evaluates the amount of utility provided by each good and bases the purchase decision on the amount of utility to be gained from each good. In following the utility maximization theory, consumers look for products that maximize utility. Utility, as shown in Equation (2), is the combination of attributes possessed by a product.

(2) $$U=f(x_{1},x_{2},x_{3}.....,x_{n})$$

*U* in this case is a function of attribute levels (*x*_1_,…,*x_n_*) and considered to be the utility or satisfaction gained from the meat. Our model focused on the attributes of the meat: tenderness, juiciness, and flavor. These variables add to satisfaction independently and when combined yield a utility.

### 2.6. Statistical Analysis

#### 2.6.1. General Model

In an effort to study the effects of beef attributes on the consumer preference, we developed a model that accounts for chosen effects and attributes. Statistical analysis of the models was performed in SAS (SAS Inst. Inc., Cary, NC, USA). The natural logs of the independent variables were used in the general model, consistent with the expectation of diminishing marginal utility. Our base model for understanding the consumer preference structure is given in Equation (3):

(3) Y=f(Tenderness,Juiciness,Flavor)

where *Y* is consumer satisfaction.

Two methods were used to evaluate the interaction of the attributes with consumer satisfaction. The reason for using two different methods was because the data presented two measurements of satisfaction. Each of the two measurements required a method that was specific to its characteristics.

#### 2.6.2. Overall Satisfaction Model

The first method that we used to approach consumer satisfaction was an Ordinary Least Squares (OLS) model. This model was used in conjunction with the continuum satisfaction measurement. The random utility model provided a direct relationship between the satisfaction and the attributes. It was applied to the general model to determine the impact of tenderness, juiciness, and flavor on overall satisfaction. For this application our general model was fitted for OLS estimation (Equation (4)).

(4) $$Y=\beta_{0}+\beta_{1}\ln Tenderness+\beta_{2}\ln Juiciness+\beta_{3}\ln Flavor+\varepsilon$$

Both the conditional logit and OLS model served specific purposes aimed at understanding the consumer preference structure. 

To evaluate if the origin of beef had any impact on consumer satisfaction, a fixed effects model was used preliminarily to determine if there was a statistical difference in the origins. Once the statistical importance of the grouping had been determined, the base model was applied to each significant grouping. 

Previous studies suggest that demographic characteristics have a substantial impact on beef preferences. The demographic characteristics of the consumers were initially dealt with by following the same procedure as Lusk and Fox [6], which involved dividing the demographical classes into two groups forming a high and low group. A dummy variable was assigned to the variable grouping in the common form of assigning the high grouping a value of one and the lower grouping a value of zero. The dummy variable was placed in the OLS regression creating a fixed effects model. If the dummy variable was statistically significant, it was assumed the two demographic categories had unique effects on the consumers’ satisfaction. 

To determine the attribute levels present, each discrete level was labeled as the attribute intensity model. This model identified statistically common expectations for quality levels that together result in satisfaction levels. This method was designed on the theory that the variance between the levels was changing. Therefore, the model cannot be simply evaluated as a simple linear regression. 

#### 2.6.3. Conditional Logit Model

The second method used was a conditional logit model. This model was used with the discrete levels of satisfaction. The discrete levels of satisfaction presented a unique problem to the model. The use of panel data allowed us to assume that the variances between the discrete levels were not uniform. Prior studies have used ordered probit, logit, and multinomial logit models to evaluate the equation. These models, however, do not account for the variance present between levels. The conditional logit model attempts to solve the issue with variance by breaking up the continuous discrete variable to account for the variance present between levels. A discrete choice can be evaluated by many statistical procedures, the most common being a form of logit modeling. The conditional logit model evaluated two consecutive discrete levels by evaluating both as a logit model. The value of one was given to the higher level and the value of zero was given to the lower level, thereby forming a simple logit model. The conditional logit model produced an odds ratio representing the probability of moving to the next discrete level with a one unit increase in the attribute. This model was specified similarly to the random utility model, but the dependent variable was in the form of a logit model of satisfaction states (Equation (5)).

(5) $$Y=\beta_{0}+\beta_{1}Tenderness+\beta_{2}Juiciness+\beta_{3}Flavor+\varepsilon$$

This model told us the probability of increasing the satisfaction of the consumer by a change in the attribute structure. This was different from the other model in many ways. The conditional logit model provided a probability of increasing consumer satisfaction, but the random utility model demonstrated the overall satisfaction based on certain attributes; however, it does not describe how to increase satisfaction. The conditional logit model illustrated how the influence of each attribute changes satisfaction. Both the conditional logit and OLS model served specific purposes aimed at understanding the consumer preference structure. 

## 3. Results and Discussion

Throughout the results the parameter estimates were considered to be the influence on consumer satisfaction unless otherwise noted. All values were positive unless a negative sign was present. Standard errors in the results were corrected for heteroskedasticity and were considered to be the robust standard errors.

### 3.1. Overall Satisfaction Model

The overall satisfaction model was the most common model used in this study. The model was adapted to examine the attribute makeup of satisfaction for origins, demographics, and discrete satisfaction levels. The following section explores the results from the various random utility models used. 

The first objective and use of the random utility model was to determine the impact of the three palatability attributes on consumer satisfaction. In order to determine the impact of the attributes on satisfaction, the semilog base model was evaluated. This model produced a high R^2^ and a moderate Residual Standard Deviation (RSD) (Table 2). By common convention, an RSD that is less than 10 is desired. Our RSD was larger than ten for some models, but it was never more than 12. 

The parameter estimates generated by the model were considered to be the impact on the continuum measurement of satisfaction, but since a semilog model was used, the estimates were transformed to represent their relationship with satisfaction. The transformation is explained as a one percent increase in any of the attributes results in a change in satisfaction. For example, a one percent increase in flavor resulted in the increase of overall satisfaction by 0.56 units. By the same transformation, a one percent increase in tenderness increased the overall satisfaction by 0.24 units, and a one percent increase in juiciness increased overall satisfaction by 0.15 units. A unit of satisfaction was represented by a one point change on the continuum scale. 

The parameter estimates indicate that flavor had the largest impact on consumer satisfaction. It was the assumption of our model that all variables were exogenous; however, endogeneity of a variable could arise, creating potential problems. These three variables were the only measures used by the survey to measure consumer satisfaction, so we assumed that they were exogenous. 

The level of importance to the consumer was depicted as (1) flavor, (2) tenderness, and (3) juiciness, with number one being the most important. The estimates suggested that flavor had a 58% greater (*p* < 0.05) impact on satisfaction than tenderness. The impact of tenderness on satisfaction was 43% greater (*p* < 0.05) than the impact of juiciness. 

O’Quinn et al. [22] determined the relative contribution of each trait to overall liking by using multivariate regression, ultimately suggesting that flavor contributed the most (49.4%), followed by tenderness (43.4%), and juiciness (7.4%). They also reported that no single palatability trait was the most important, as beef palatability was dependent upon the acceptance of all three traits. Flavor had a greater contribution to beef overall liking [22], but not to the extent that we observed in the current findings. Previous work has shown strong relationships between beef flavor and overall acceptability or liking [23,24,25]. In fact, flavor was the most highly correlated trait to overall liking as opposed to juiciness or tenderness. The current results were not unexpected as the previous reports of beef eating quality for US consumers align with these coefficients for grain-fed beef [26,27] and grass-fed beef [28,29,30].

### 3.2. Impacts of Origin on Satisfaction

The next objective of the study was to determine if beef origin (the source country and cattle finishing system) had any impact on consumer preferences. For the preliminary determination, a fixed effects model was used. The results of the model showed that origin had an impact (*p* < 0.05) on consumer satisfaction (Table 3). The model was run using each origin as the base; therefore, the R^2^ and the RSD are the same for each base. This was done to ensure that the rankings were consistent. USDA Select and USDA Choice had statistically different impacts on satisfaction, but the two Australian finishing systems (grass-fed or grain-fed) had similar impacts on satisfaction. More importantly, we found that Australian beef had a statistically different impact than the USDA cuts on satisfaction. The ranking produced by the model align with previous results [20,25,30,31] that consumers prefer the flavor of domestic beef, especially when compared to international grass-fed beef.

Estimates of the pairwise model showed that US beef produced greater (*p* < 0.05) consumer satisfaction than the Australian grass-fed beef. One of the biggest differences in the origins was the consistently higher estimates for flavor in the US beef (Table 4). The parameter estimates revealed that the flavor of the USDA Choice was approximately 12% greater than Australian grass-fed and 3% greater than Australian grain-fed beef. Consumers were 2% more responsive to the flavor of Australian grain-fed beef than USDA Select, but responded more favorably to the tenderness of USDA Select.

The impact of origin was also evaluated with the attribute intensity model (Table 5). For USDA Choice beef and the Australian grain-fed beef, tenderness did not impact (*p* > 0.05) satisfaction at the “premium quality” level, but tenderness was a significant attribute affecting (*p* < 0.05) satisfaction of the USDA Select samples. Tenderness, juiciness, and flavor impacted (*p* < 0.05) satisfaction at all other levels across all origins. Results of this model showed that the flavor in Australian grass-fed beef was not as strong as the US beef at the “better than everyday quality” level, and Australian grain-fed beef was not as strong as the US beef especially at the “premium quality” level. At the premium level, USDA Choice flavor was 29% greater than Australian beef, while the USDA Select flavor was 42% higher than Australian grain-fed beef. For USDA Choice beef and the Australian grain-fed beef, tenderness did not impact (*p* > 0.05) satisfaction at the “premium quality” level, but tenderness was a significant attribute affecting (*p* < 0.05) satisfaction of the USDA Select samples. 

We also used this model to understand the differences present in the levels between product origin (source country and finishing system). Results suggested that people were extremely decisive about what beef samples they deem “unsatisfactory.” Attribute estimates for “unsatisfactory” and “good everyday quality” beef seemed to be fairly similar across the product origins. The results also showed that consumers were less decisive about what they deem “premium quality,” indicated by large standard errors. 

### 3.3. Impact of Demographics

Our last use of our random utility model was to determine the impact that demographic variables had on consumer satisfaction. The four demographic variables evaluated in this model were age, income, education, and gender, as each has been shown to impact consumer perception [4,19]. As seen in Table 6, education did not impact satisfaction (*p* > 0.05) in the overall model, but age, income, and gender influenced (*p* < 0.05) satisfaction. 

A fixed effects model was used to determine the significance of the demographical groupings and to determine if any of the demographical effects carried over into the origins (Table 6). The only observed significance occurred in the age groups in the Australian beef and income groups in the USDA Choice. Because of the large scope of this study, these interactions were not investigated further. 

Once we had determined that income, gender, and age impacted satisfaction, we applied the previous models. A pairwise comparison of the groupings was performed, followed by the attribute intensity model. These models allowed us to understand the reason for the differences between groups. 

#### 3.3.1. Gender

The fixed effects model suggested that the females were going to be less responsive to changes in the attributes (Table 7). The pairwise comparison of gender indicated that males were more responsive to a change in all attributes than females. The most distinct difference was that males were 7% more responsive to a change in tenderness.

Bonny et al. [32] reported demographic differences of European consumer scores for beef eating quality. Notably, men scored beef samples more favorably than women in general, but this trend varied between countries (Ireland, Northern Ireland, and Poland) and between palatability traits. Only Irish and Northern Irish males scored beef juicier with greater flavor liking than females, while only Polish males rated tenderness higher than females. Kubberød et al. [33] also reported men score meat more favorably than females, which was likely attributed to their more positive attitude towards red meat. 

The attribute intensity model showed that males and females disagree on what cut of meat should be “unsatisfactory.” At the “unsatisfactory” level, males were 13% more responsive to tenderness while females were 11% more responsive to juiciness (Table 8). A noticeable difference also occurred at the “better than everyday quality” level, where males were 24% more responsive to juiciness while females were 12% more responsive to tenderness. Males and females seemed to agree on the attribute make-up for a “premium quality” cut of beef. 

#### 3.3.2. Age

The next demographical variable presented was age. The fixed effects model suggested that older consumers were more responsive to changes in the attributes. The outcome of the pairwise comparison showed that the two age groupings were relatively close in the preferences for the attributes (Table 9). One distinct difference was that younger participants were more responsive to changes in juiciness by almost 6%. 

Bonny et al. [32] observed a small negative relationship between age and tenderness in France and Poland, and with age and juiciness in Ireland, Northern Ireland, and Poland. However, tenderness scores increased with age in Northern Ireland. Age can also influence red meat purchase intent of Canadian consumers [34]. Quagrainie et al. [34] reported that people aged 30 years old or younger were more likely to purchase meat products (specifically high quality pork, high quality beef, or ground beef) than older consumers.

The next area evaluated was the attribute intensity for each discrete level in the two age groups. Both groups were commonly decisive as to what cuts of meat were “unsatisfactory.” Once again, as the satisfaction level increased the standard error increased, suggesting that the consumers were uncertain about the personal classification of “premium quality.”

The older group had a greater response to the flavor attribute than the younger age group at all levels. This was most evident at the “good everyday quality” and “premium quality” levels (Table 10). At these levels, the older group was 7% more responsive to changes in flavor. There was less variation present in the older group’s estimates suggesting they have developed their quality expectations more thoroughly. Inconsistencies between the age groups were evident at the “better than everyday quality” level, where older consumers were 38% more responsive to tenderness, but younger consumers were 18% more responsive to juiciness. The younger panelists were more responsive to juiciness in the top three levels by a minimum of 16%.

#### 3.3.3. Income

The last demographic examined was income. The fixed effects model suggested that the lower income group was more responsive to a change in attributes. The results from the pairwise comparison illustrated that higher income consumers were more responsive to a change in tenderness, by nearly 11%, while the lower income group was more responsive to juiciness and flavor (Table 11).

Bonny et al. [32] reported income level with respect to country had very little effect on consumer scores for beef eating quality. Only juiciness was influenced by income level [35]. Likewise, Hwang et al. [35] reported demographic characteristics had minimal impact on beef sensory scores given by Korean and Australia consumers. 

The attribute intensity model showed there were inconsistencies about the attributes that qualify as an “unsatisfactory” cut of beef (Table 12). The higher income group was 15% more responsive to tenderness at this level, but the lower income group was 10% more responsive to juiciness. The biggest factor separating the higher income consumers from the lower income consumers seemed to be the difference in tenderness preferences at all levels. The lower income group was more responsive to flavor at all levels except for the “premium quality” level. The lower income group was 10% more responsive to juiciness at the “premium quality” level. The variation for the lower income group was greater, suggesting that the higher income group had a more defined perception of satisfaction. 

### 3.4. Attribute Intensity Model

In an effort to determine the impacts of the attributes at each satisfaction level, we applied the base model to each discrete satisfaction level. To ensure the integrity of this model, we had to first guarantee that the satisfaction levels were statistically different from each other. A fixed effects model was used to show that the levels were indeed different. The fixed effects model showed that the discrete levels follow their ordinal ranking as expected. The pairwise comparison of all the levels allowed us to determine the difference in the levels, implying that a change in attributes at the “premium quality” level had a 63% greater impact on satisfaction than the same change at the “unsatisfactory” level. This discrepancy gap became narrower as satisfaction level elevated. For example, the change in parameter estimates between “premium quality” and “better than everyday quality” was only 2%. 

The base model was applied to each discrete level for the attribute intensity model (Table 13). This model also showed how the attributes’ impacts vary between levels. Results showed that once again the flavor attribute garnered the largest parameter estimate in each of the levels. Juiciness appeared to be the attribute more responsible for change between “premium quality” and “better than everyday quality.” The parameter estimate for juiciness increased by 25% between these two levels, while the other attributes decreased a small amount, suggesting diminishing marginal utility was present. 

Results indicated that consumers were not decisive when classifying beef as “premium quality,” as shown through the increase in standard error from “better than everyday quality” to “premium quality.” On the other hand, consumers do not have a problem classifying beef as “unsatisfactory,” as indicated by the smaller standard error and greater difference in parameter estimates at these levels. Our model demonstrated that the parameter estimates changed about 52% between “unsatisfactory” and “everyday quality” levels. This was the largest difference between levels found in our model. 

### 3.5. Conditional Logit Model

The conditional logit model was used to determine the probability of changing discrete levels with an attribute change. This model was designed to compare two consecutive levels as a logit model. The higher level was given a value of one, while the lower level was given a value of zero. The point estimate produced by the model indicated the probability of changing satisfaction levels with a one unit change in the attribute. The point estimate was the exponentiated value of the parameter estimates. The tables included in this section show the parameter estimates along with the point estimates. 

This model was first applied to the overall model. Previously in the conceptual model we assumed that the variables would exhibit a diminishing marginal utility. The results shown in Table 14 illustrate the diminishing marginal utility of flavor, as indicated by the decreasing point estimate as the satisfaction level increased. 

Table 14 shows the consumers exhibited a linear preference structure between levels, which translated to an equal distribution of attributes when changing between levels. For example, the probability for a change from “better than everyday quality” to “premium quality” consisted of 34% flavor, while the change from “unsatisfactory” to “everyday quality” was 35%. 

Results of the conditional logit model suggested that an increase in flavor created the largest increase in the probability of moving from “unsatisfactory” to “good everyday quality.” However, for samples classified as “good everyday quality” or higher, flavor played a minimal role in increasing the probability of moving to the next level. This model also showed that tenderness had the largest impact on the probability of increasing to “premium quality” from “better than everyday quality.”

The conditional logit model was also used to evaluate the impact of origin on consumer satisfaction (Table 15). Flavor was the most important factor for increasing satisfaction at all levels and origins, except for Australian grain-fed and USDA Select beef moving from “better than everyday quality” to “premium quality.” As quality level increased more emphasis shifted to tenderness, but flavor was still the driving force behind satisfaction in most instances. 

## 4. Conclusions

Perhaps the most important finding of this study was that flavor was the largest contributor to consumer satisfaction. This finding was consistent throughout all the models. In the base model, flavor represented 59% of the satisfaction rating. Additionally, results indicated domestic beef was preferred over Australian beef by US consumers. The use of our base model showed that US beef was preferred in all categories, but primarily due to flavor. The flavor generated by the US beef was at least 2% greater in the overall satisfaction model. Americans are accustomed to eating domestic grain-fed beef and may have acquired a preference for the flavor of US beef over beef from other countries. In addition, differences in feeding practices between Australia and the US may impact the flavor of beef. 

Another important finding of the study was the impact of the demographic variables of age, income, and gender on satisfaction. The older group generally placed more emphasis on tenderness, while younger people preferred a juicier beef. Males were more responsive than females for all attributes, especially tenderness. Those with higher income were more responsive to tenderness for all quality levels, but the lower income group was more responsive to juiciness. In conclusion, this approach effectively integrated beef source (country and cattle finishing system) and sociodemographic factors of US consumers to generate insights into the drivers of beef satisfaction.

## Figures and Tables

**Table 1:** Consumer demographics and responses to beef preference statements. Reported as percentages of consumers (*n* = 1440; 480/city).

	Overall	Lubbock	Washington DC	Phoenix
**Age**				
20–30	34.5	32.9	49.3	21.4
31–40	21.1	17.2	16.6	29.6
41–50	26.7	30.6	17.1	32.2
51–60	16.0	19.1	12.0	16.8
>60	1.7	0.2	5.0	0.0
**Income**				
<USD 20,000	12.0	16.0	12.7	7.2
USD 20,000–50,000	28.8	27.6	34.1	24.8
USD 51,000–75,000	24.5	26.3	20.2	26.8
USD 76,000–100,000	15.8	16.5	14.4	16.4
>USD 100,000	19.0	13.5	18.6	24.8
**Gender**				
Male	49.9	44.5	55.5	49.7
Female	50.1	55.5	44.5	50.3
**Education**				
Non-High School Graduate	2.7	1.6	1.6	2.6
High School Graduate	9.8	9.7	9.7	9.3
Some College/Technical School	28.1	32.0	32.0	31.4
College Graduate	36.6	35.4	35.4	34.6
Post Graduate	22.8	21.2	21.2	22.1
**Preferred Doneness**				
Blue	0.1	0.2	0.2	0.0
Rare	3.2	3.4	4.2	1.9
Medium Rare	26.2	27.6	29.3	21.4
Medium	30.2	32.0	28.7	29.6
Medium Well	30.0	31.5	24.5	33.5
Well Done	10.3	5.3	11.7	13.6
**Statement**				
I enjoy red meat. It’s an important part of my diet.	46.7	56.3	44.6	39.1
I like red meat well enough. It’s a regular part of my diet.	37.9	32.9	38.7	42.0
I do eat some red meat although, but it wouldn’t worry me if I didn’t.	12.4	8.8	13.5	15.0
I rarely/never eat red meat.	3.0	2.0	3.2	3.9
**Regular Purchaser of Beef**				
Yes	70.7	72.1	68.7	71.3
No	29.3	27.9	31.3	28.7
**Grade of Beef Most Commonly Purchased ^a^**				
USDA Prime	14.5	9.0	18.9	15.8
USDA Choice	51.2	55.4	47.7	50.6
USDA Select	11.4	14.1	8.6	11.4
Other	22.9	21.5	24.9	22.2
**Important Palatability Trait for Roasts**				
Flavor	40.2	38.3	38.5	42.7
Tenderness	47.8	49.2	48.5	46.3
Juiciness	12.1	12.5	13.0	10.9
**Important Palatability Trait for Steaks**				
Flavor	39.4	38.2	40.9	38.6
Tenderness	46.8	45.9	46.7	47.3
Juiciness	13.8	15.8	12.4	14.1

^a^ USDA = United States Department of Agriculture.

**Table 2:** Impacts of attributes on overall satisfaction using a random utility model.

Variables	Estimates	SE
Intercept	−105.28 *	1.551
Tenderness	23.99 *	0.751
Juiciness	15.17 *	0.782
Flavor	56.47 *	1.050

*n* = 9357; R^2^ = 0.77; RSD = 11.15. * Denotes variables significant at *p* < 0.05.

**Table 3:** Fixed effects model for origin impact on consumer satisfaction.

Base Origin	Variables	Estimates	SE	R^2^ (RSD)
Australian Grass	Intercept	−105.73 *	1.548	0.78
	Tenderness	23.85 *	0.750	(11.47)
	Juiciness	15.18 *	0.784	
	Flavor	56.30 *	1.049	
	USDA Select	1.16 *	0.381	
	USDA Choice	2.09 *	0.378	
	Australian Grain	0.42	0.363	

* Denotes variables significant at *p* < 0.05.

**Table 4:** Comparisons of origins and attribute effects.

Origin	Variables	Estimates	SE	R^2^ (RSD)	*n*
Australian Grass	Intercept	−103.00 *	3.15	0.78	1511
	Tenderness	27.12 *	1.70	(11.54)	
	Juiciness	14.76 *	1.95		
	Flavor	51.82 *	2.33		
Australian Grain	Intercept	−103.53 *	2.50	0.78	2998
	Tenderness	21.76 *	1.27	(11.50)	
	Juiciness	15.27 *	1.24		
	Flavor	57.23 *	1.70		
USDA Select	Intercept	−101.20 *	3.12	0.77	2410
	Tenderness	23.25 *	1.48	(11.78)	
	Juiciness	14.04 *	1.51		
	Flavor	56.06 *	2.30		
USDA Choice	Intercept	−112.26 *	1.97	0.79	2411
	Tenderness	24.91 *	1.02	(11.34)	
	Juiciness	16.83 *	1.05		
	Flavor	58.82 *	1.39		

* Denotes variables significant at *p* < 0.05.

**Table 5:** Comparison of attribute intensity within different origins.

	USDA Choice Beef				USDA Select Beef				Australian Grain-Fed Beef				Australian Grass-Fed Beef			
			R^2^				R^2^				R^2^				R^2^	
Variables	Est.	SE	(RSD)	*n*	Est.	SE	(RSD)	*n*	Est.	SE	(RSD)	*n*	Est.	SE	(RSD)	*n*
**Unsatisfactory**																
Intercept	−38.65 *	2.974	0.58	424	−29.60 *	2.534	0.54	499	−37.53 *	2.313	0.59	701	−36.96 *	3.205	0.56	390
Tenderness	10.56 *	1.621	(9.87)		8.32 *	1.497	(9.09)		10.37 *	1.181	(8.88)		11.81 *	1.588	(8.85)	
Juiciness	7.39 *	1.743			6.51 *	1.502			5.59 *	1.284			7.63 *	1.812		
Flavor	29.12 *	1.699			26.04 *	2.083			29.88 *	1.316			25.59 *	1.609		
**Good Everyday Quality**																
Intercept	−109.88 *	11.570	0.60	987	−98.05 *	4.5013	0.62	1081	−107.59 *	4.248	0.62	1330	−105.48 *	5.716	0.60	652
Tenderness	18.40 *	2.542	(9.98)		19.07 *	1.745	(9.52)		17.63 *	1.909	(9.68)		23.89 *	2.515	(9.57)	
Juiciness	14.56 *	2.703			14.01 *	1.761			13.17 *	1.582			9.94 *	2.501		
Flavor	63.54 *	4.566			56.44 *	2.766			63.88 *	2.567			59.74 *	3.440		
**Better than Everyday Quality**																
Intercept	−152.52 *	13.663	0.62	667	−162.70 *	10.264	0.62	562	−134.02 *	18.178	0.56	705	−116.25 *	30.604	0.43	331
Tenderness	22.07 *	5.635	(7.13)		32.72 *	4.943	(6.79)		22.79 *	4.470	(7.95)		30.96 *	7.960	(9.58)	
Juiciness	11.12 *	3.795			11.50 *	2.412			15.36 *	3.557			15.43 *	6.914		
Flavor	89.98 *	9.158			84.06 *	5.320			74.79 *	9.217			56.43 *	17.274		
**Premium Quality**																
Intercept	−148.51 *	40.662	0.61	333	−179.27 *	16.164	0.82	268	−74.71 *	33.775	0.42	258	−216.42 *	18.378	0.81	136
Tenderness	32.83	21.819	(4.93)		30.77 *	9.330	(4.68)		16.76	13.319	(7.64)		28.95 *	12.413	(4.65)	
Juiciness	13.74 *	6.128			14.66 *	4.342			14.34 *	8.301			31.68 *	8.205		
Flavor	76.78 *	13.395			93.58 *	5.652			54.29 *	17.262			87.26 *	16.206		

* Denotes variables significant at *p* < 0.05. Est. denotes Estimates.

**Table 6:** Demographic effects on satisfaction.

	Overall Model			USDA Choice Beef			USDA Select Beef			Australian Beef		
			R^2^			R^2^			R^2^			R^2^
Variables	Estimates	SE	(RSD)	Estimates	SE	(RSD)	Estimates	SE	(RSD)	Estimates	SE	(RSD)
Intercept	−116.65 *	1.256	0.84	−126.03	2.757	0.85	−113.85 *	2.529	0.82	−113.36 *	1.693	0.83
Tenderness	25.80 *	0.568	(9.66)	27.72 *	1.250	(9.16)	25.02 *	1.105	(9.84)	25.15 *	0.775	(9.77)
Juiciness	16.01 *	0.608		17.32 *	1.447		14.56 *	1.076		16.25 *	0.853	
Flavor	60.98 *	0.812		63.94 *	1.784		61.53 *	1.689		58.97 *	1.077	
Income	−0.78 *	0.218		−1.27 *	0.418		−0.69	0.436		−0.61	0.315	
Age	0.69 *	0.209		0.24	0.384		0.39	0.424		1.08 *	0.306	
Education	0.37	0.256		−0.63	0.481		−0.15	0.488		−0.29	0.382	
Gender	−0.52 *	0.202		−0.67	0.375		−0.63	0.406		−0.35	0.295	

* Denotes variables significant at *p* < 0.05.

**Table 7:** Pairwise comparisons of gender on consumer satisfaction.

	Male				Female			
Variables	Estimates	SE	R^2^ (RSD)	*n*	Estimates	SE	R^2^ (RSD)	*n*
Intercept Tenderness Juiciness Flavor	−121.32 * 26.84 * 16.29 * 61.77 *	1.847 0.840 0.914 1.239	0.83 (9.42)	4650	−114.42 * 25.02 * 15.80 * 60.42 *	1.608 0.766 0.817 1.069	0.84 (9.92)	4580

* Denotes variables significant at *p* < 0.05.

**Table 8:** Comparison of attribute intensity for gender by satisfaction level.

	Male				Female			
Variables	Estimates	SE	R^2^ (RSD)	*n*	Estimates	SE	R^2^ (RSD)	*n*
**Unsatisfactory**								
Intercept Tenderness Juiciness Flavor	−51.09 * 13.65 * 8.25 * 33.92 *	1.936 0.895 0.977 1.180	0.70 (7.27)	833	−49.63 * 11.92 * 9.13 * 33.87 *	1.574 0.824 0.874 0.919	0.71 (7.44)	962
**Good Everyday Quality**								
Intercept Tenderness Juiciness Flavor	−108.02 * 21.67 * 14.59 * 58.95 *	2.819 1.129 1.093 1.785	0.67 (8.33)	2063	−112.18 * 22.33 * 13.46 * 62.03 *	2.995 1.175 1.093 1.843	0.70 (8.72)	1924
**Better than Everyday Quality**								
Intercept Tenderness Juiciness Flavor	−158.18 * 24.97 * 16.54 * 84.47 *	7.659 2.766 1.993 4.681	0.66 (6.72)	1148	−149.91 * 27.96 * 12.51 * 81.00 *	7.194 2.932 1.893 3.645	0.63 (7.30)	1158
**Premium Quality**								
Intercept Tenderness Juiciness Flavor	−199.92 * 39.71 * 14.20 * 95.54 *	8.465 5.822 3.535 6.246	0.76 (4.77)	602	−188.59 * 39.68 * 13.86 * 90.14 *	17.188 9.549 3.506 9.382	0.75 (4.88)	535

* Denotes variables significant at *p* < 0.05.

**Table 9:** Pairwise comparisons of age groups for consumer satisfaction.

	20–40 Years Old				41–60 Years Old			
Variables	Estimates	SE	R^2^ (RSD)	*n*	Estimates	SE	R^2^ (RSD)	*n*
Intercept Tenderness Juiciness Flavor	−118.25 * 25.58 * 16.48 * 61.11 *	1.6826 0.7528 0.8511 1.1169	0.83 (9.75)	5113	−116.54 * 26.34 * 15.52 * 60.61 *	1.716 0.871 0.874 1.181	0.84 (9.60)	4117

* Denotes variables significant at *p* < 0.05.

**Table 10:** Comparison of attribute intensity for age groups by satisfaction level.

	20–40 Years Old				41–60 Years Old			
Variables	Estimates	SE	R^2^ (RSD)	*n*	Estimates	SE	R^2^ (RSD)	*n*
**Unsatisfactory**								
Intercept Tenderness Juiciness Flavor	−50.18 * 12.90 * 8.85 * 33.28 *	1.577 0.800 0.868 0.949	0.71 (7.25)	964	−50.42 * 12.45 * 8.60 * 34.62 *	1.903 0.926 0.971 1.095	0.70 (7.48)	831
**Good Everyday Quality**								
Intercept Tenderness Juiciness Flavor	−108.81 * 21.56 * 15.39 * 58.87 *	2.901 1.104 1.105 1.708	0.67 (8.67)	2264	−112.10 * 22.39 * 12.41 * 62.82 *	2.884 1.223 1.089 1.926	0.71 (8.30)	1723
**Better than Everyday Quality**								
Intercept Tenderness Juiciness Flavor	−147.16 * 23.67 * 15.29 * 81.15 *	7.104 2.489 1.842 3.960	0.64 (7.12)	1296	−166.06 * 32.65 * 12.60 * 84.69 *	6.972 3.439 2.041 3.837	0.66 (6.86)	1010
**Premium Quality**								
Intercept Tenderness Juiciness Flavor	−194.23 * 41.33 * 15.52 * 89.77 *	13.979 9.280 3.523 10.136	0.77 (4.88)	584	−195.41 * 38.28 * 13.06 * 95.72 *	11.673 6.771 3.338 6.127	0.75 (4.76)	553

* Denotes variables significant at *p* < 0.05.

**Table 11:** Pairwise comparisons of income groups on consumer satisfaction.

	USD 0–50,000				USD 50,000 and above			
Variables	Estimates	SE	R^2^ (RSD)	*n*	Estimates	SE	R^2^ (RSD)	*n*
Intercept Tenderness Juiciness Flavor	−116.38 * 24.26 * 16.08 * 62.12 *	1.536 0.732 0.745 1.017	0.82 (10.17)	3762	−117.96 * 26.96 * 15.85 * 60.16 *	1.985 0.897 1.037 1.330	0.85 (9.32)	5468

* Denotes variables significant at *p* < 0.05.

**Table 12:** Comparison of attribute intensity for income groups by satisfaction level.

	USD 0–50,000				USD 50,000 and above			
Variables	Estimates	SE	R^2^ (RSD)	*n*	Estimates	SE	R^2^ (RSD)	*n*
**Unsatisfactory**								
Intercept Tenderness Juiciness Flavor	−49.27 * 11.63 * 9.31 * 33.98 *	1.973 1.003 1.112 1.222	0.70 (7.82)	670	−50.88 * 13.34 * 8.38 * 33.85 *	1.549 0.742 0.790 0.884	0.72 (7.07)	1125
**Good Everyday Quality**								
Intercept Tenderness Juiciness Flavor	−108.57 * 21.08 * 13.34 * 61.44 *	3.453 1.278 1.392 2.118	0.66 (9.27)	1656	−110.69 * 22.67 * 14.22 * 59.84 *	2.502 1.050 0.913 1.569	0.71 (7.93)	2331
**Better than Everyday Quality**								
Intercept Tenderness Juiciness Flavor	−151.60 * 25.36 * 14.07 * 83.20 *	8.210 3.301 2.226 4.609	0.62 (7.40)	961	−154.53 * 27.09 * 14.55 * 82.17 *	6.639 2.493 1.748 3.733	0.67 (6.73)	1345
**Premium Quality**								
Intercept Tenderness Juiciness Flavor	−188.06 * 39.96 * 15.27 * 88.31 *	13.819 9.022 4.260 10.890	0.79 (5.15)	472	−203.17 * 40.58 * 13.73 * 96.69 *	11.300 6.437 2.961 5.248	0.74 (4.56)	553

* Denotes variables significant at *p* < 0.05.

**Table 13:** Comparison of attribute levels of corresponding satisfaction levels.

Satisfaction Level	Variables	Estimates	SE	R^2^ (RSD)	*n*
Unsatisfactory	Intercept Tenderness Juiciness Flavor	−35.75 * 10.48 * 6.37 * 27.92 *	1.343 0.727 0.774 0.850	0.57 (9.78)	2018
Good Everyday Quality	Intercept Tenderness Juiciness Flavor	−104.42 * 19.26 * 13.20 * 60.65 *	3.392 1.118 1.019 1.658	0.61 (9.73)	4063
Better than Everyday Quality	Intercept Tenderness Juiciness Flavor	−140.97 * 25.71 * 13.44 * 77.58 *	10.179 2.929 1.973 5.909	0.56 (7.78)	2273
Premium Quality	Intercept Tenderness Juiciness Flavor	−140.95 * 25.60 * 17.92 * 75.93 *	18.876 8.529 4.683 11.416	0.65 (5.91)	997

* Denotes variables significant at *p* < 0.05.

**Table 14:** Conditional logit model.

Transition	Variables	Estimates	SE	Point Estimates
Level 2 to Level 3 ^a^	Intercept Tenderness Juiciness Flavor	−4.43 * 0.04 * 0.01 * 0.08 *	0.135 0.002 0.002 0.002	1.037 1.004 1.079
Level 3 to Level 4 ^a^	Intercept Tenderness Juiciness Flavor	−8.10 * 0.04 * 0.01 * 0.06 *	0.207 0.002 0.002 0.002	1.042 1.013 1.059
Level 4 to Level 5 ^a^	Intercept Tenderness Juiciness Flavor	−11.86 * 0.06 * 0.01 * 0.05 *	0.458 0.005 0.003 0.005	1.065 1.015 1.055

* Denotes variables significant at *p* < 0.05. ^a^ Level 2 = Unsatisfactory; Level 3 = Good everyday quality; Level 4 = Better than everyday quality; Level 5 = Premium quality.

**Table 15:** Conditional logit model focusing on origin.

		Level 2 vs. Level 3 ^a^			Level 3 vs. Level 4 ^a^			Level 4 vs. Level 5 ^a^		
Origin	Variables	Estimates	SE	Point Estimates	Estimates	SE	Point Estimates	Estimates	SE	Point Estimates
Australian Grass	Intercept Tenderness Juiciness Flavor	−4.85 * 0.05 * 0.00 0.08 *	0.336 0.005 0.005 0.006	1.047 1.000 1.082	−8.17 * 0.05 * 0.01 * 0.05 *	0.534 0.005 0.005 0.006	1.048 1.015 1.052	−13.29 * 0.07 * 0.02 * 0.07 *	1.326 0.014 0.009 0.012	1.039 0.999 1.043
Australian Grain	Intercept Tenderness Juiciness Flavor	−4.50 * 0.03 * 0.00 0.08 *	0.233 0.003 0.003 0.004	1.035 1.005 1.082	−8.01 * 0.04 * 0.02 * 0.05 *	0.363 0.003 0.003 0.004	1.041 1.016 1.056	−10.82 * 0.06 * 0.02 * 0.04 *	0.797 0.009 0.006 0.008	1.061 1.022 1.038
USDA Select	Intercept Tenderness Juiciness Flavor	−4.18 * 0.04 * 0.00 0.07 *	0.256 0.004 0.004 0.005	1.039 1.000 1.076	−8.51 * 0.04 * 0.01 * 0.06 *	0.432 0.004 0.004 0.005	1.045 1.010 1.064	−11.59 * 0.06 * 0.01 * 0.05 *	0.882 0.009 0.006 0.009	1.063 1.015 1.056
USDA Choice	Intercept Tenderness Juiciness Flavor	−4.39 * 0.03 * 0.01 * 0.08 *	0.295 0.004 0.004 0.005	1.030 1.009 1.080	−7.85 * 0.04 * 0.01 * 0.06 *	0.390 0.004 0.004 0.005	1.039 1.011 1.062	−13.21 * 0.07 * 0.01 * 0.07 *	0.906 0.009 0.006 0.009	1.069 1.010 1.072

* Denotes variables significant at *p* < 0.05. ^a^ Level 2 = Unsatisfactory; Level 3 = Good everyday quality; Level 4 = Better than everyday quality; Level 5 = Premium quality.

## Supplementary Materials

The following are available online at https://www.mdpi.com/2304-8158/9/3/289/s1, Figure S1: Scatter plot illustrating the relationship between raw flavor data plotted against satisfaction. 0 = dislike flavor/overall extremely; 100 = like flavor/overall extremely, Figure S2: Scatter plot illustrating the relationship between flavor data cleaned with Cook’s Distance method plotted against satisfaction. 0 = dislike flavor/overall extremely; 100 = like flavor/overall extremely, Figure S3: Scatter plot illustrating the relationship between raw tenderness data plotted against satisfaction. 0 = dislike flavor/overall extremely; 100 = like flavor/overall extremely, Figure S4: Scatter plot illustrating the relationship between tenderness data cleaned with Cook’s Distance method plotted against satisfaction. 0 = dislike flavor/overall extremely; 100 = like flavor/overall extremely, Figure S5: Scatter plot illustrating the relationship between raw juiciness data plotted against satisfaction. 0 = dislike flavor/overall extremely; 100 = like flavor/overall extremely, Figure S6: Scatter plot illustrating the relationship between juiciness data cleaned with Cook’s Distance method plotted against satisfaction. 0 = dislike flavor/overall extremely; 100 = like flavor/overall extremely.
