# Supplementary material for: Beef Quality Preferences: Factors Driving Consumer Satisfaction
Source: Foods. 2020 Mar 4;9(3):289. doi: 10.3390/foods9030289 (PMC7143558; doi:10.3390/foods9030289)
Supplement: Supplementary file 1 [file foods-09-00289-s001.pdf]

Supplementary Figures

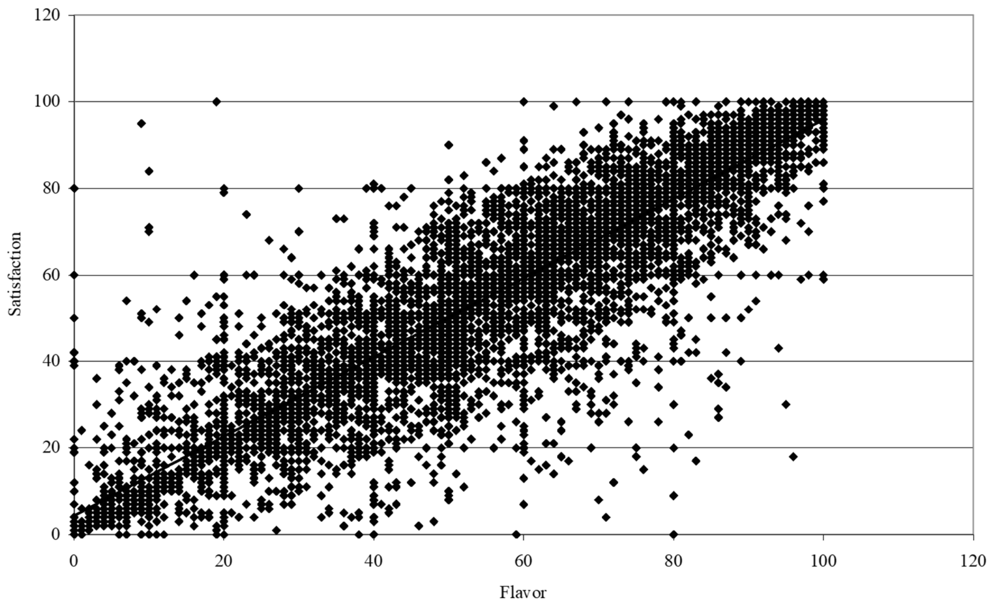

Figure S1. Scatter plot illustrating the relationship between raw flavor data plotted against satisfaction. 0 = dislike flavor/overall extremely; 100 = like flavor/overall extremely.

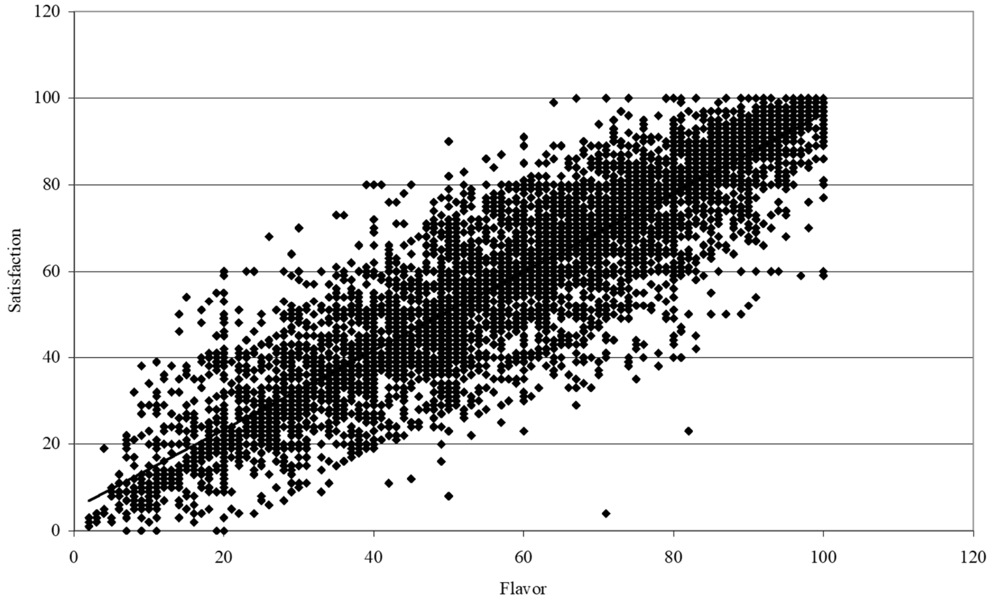

Figure S2. Scatter plot illustrating the relationship between flavor data cleaned with Cook's Distance method plotted against satisfaction. 0 = dislike flavor/overall extremely; 100 = like flavor/overall extremely.

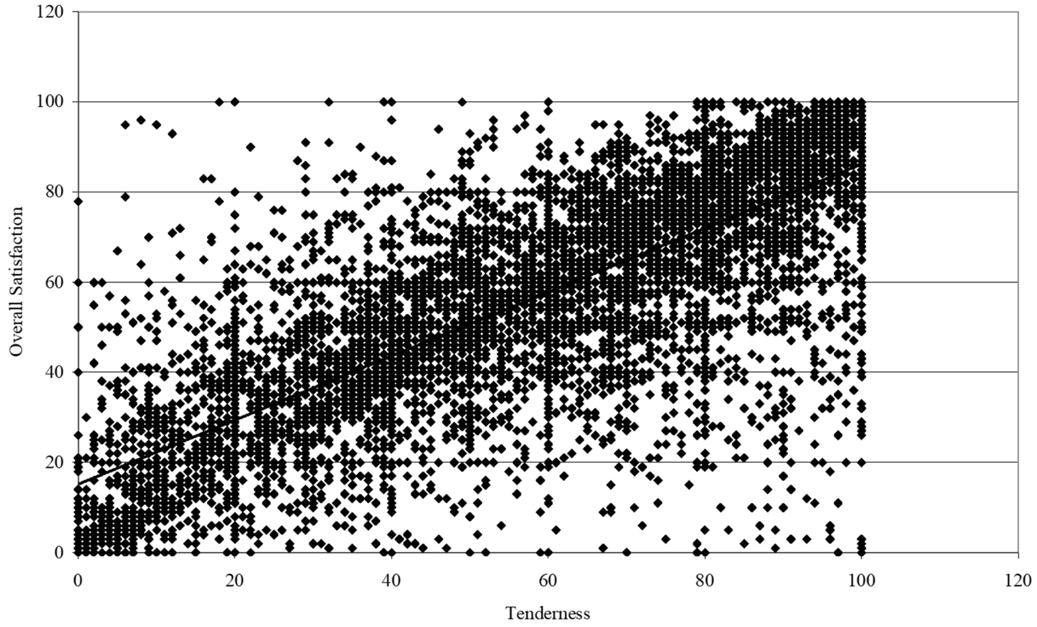

Figure S3. Scatter plot illustrating the relationship between raw tenderness data plotted against satisfaction. 0 = dislike flavor/overall extremely; 100 = like flavor/overall extremely.

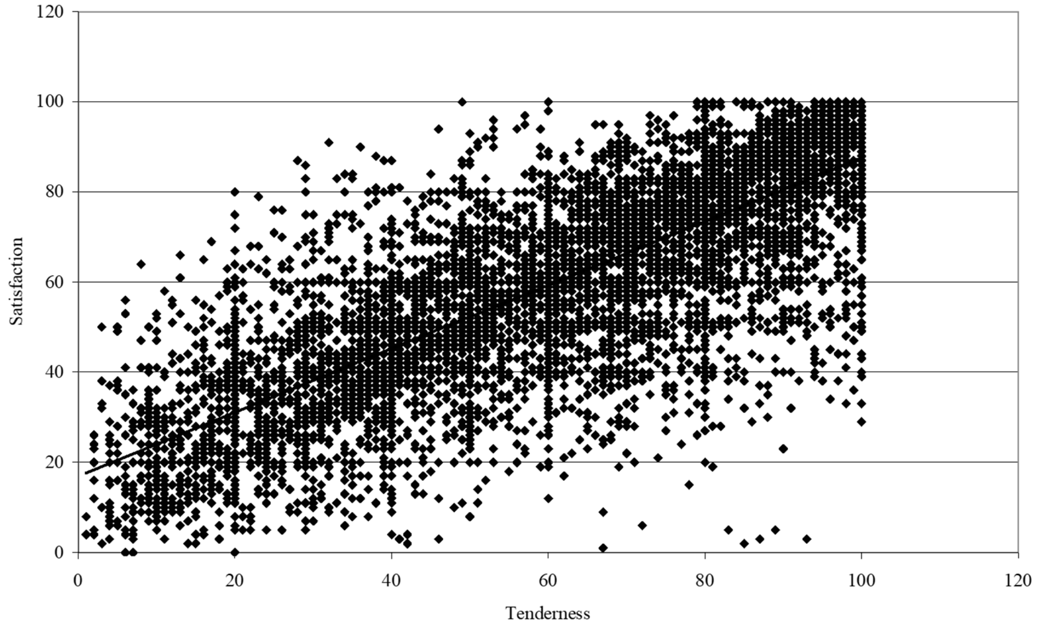

Figure S4. Scatter plot illustrating the relationship between tenderness data cleaned with Cook's Distance method plotted against satisfaction. 0 = dislike flavor/overall extremely; 100 = like flavor/overall extremely.

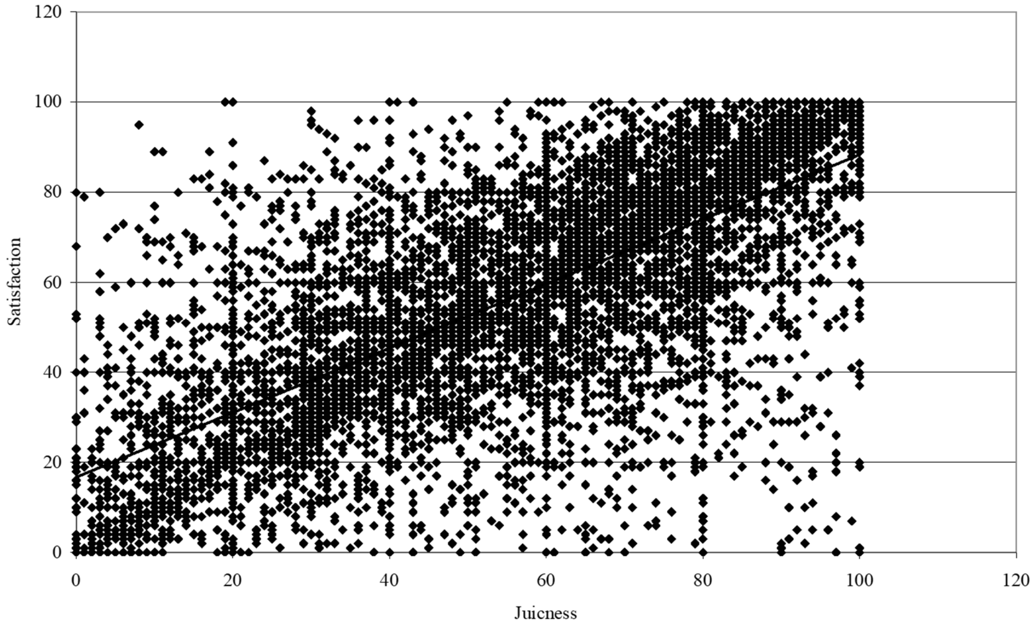

Figure S5. Scatter plot illustrating the relationship between raw juiciness data plotted against satisfaction. 0 = dislike flavor/overall extremely; 100 = like flavor/overall extremely.

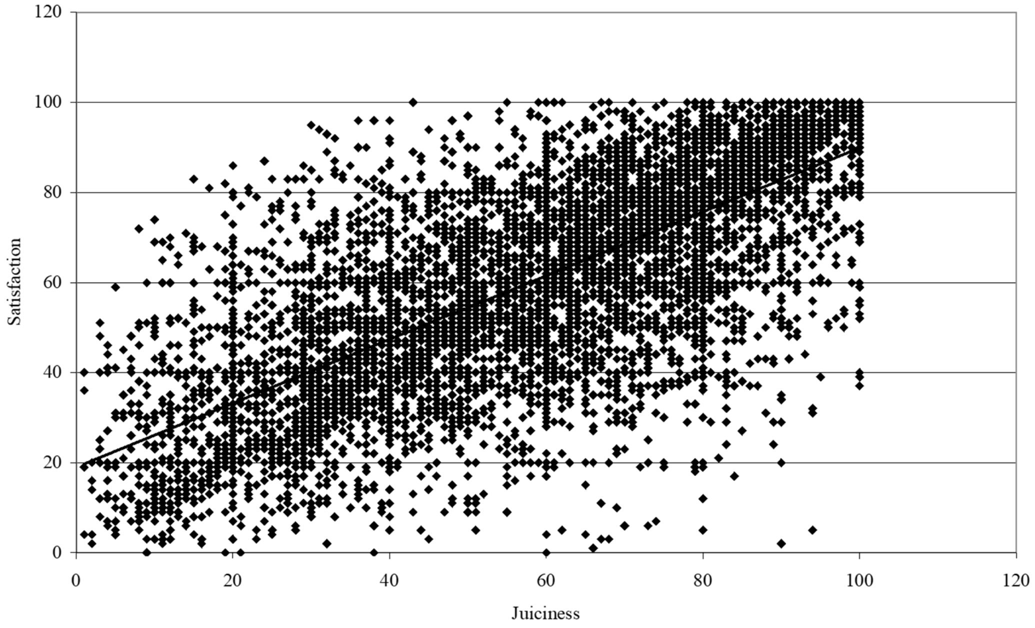

Figure S6. Scatter plot illustrating the relationship between juiciness data cleaned with Cook's Distance method plotted against satisfaction. 0 = dislike flavor/overall extremely; 100 = like flavor/overall extremely.
